# Chemical Transport Knockout for Oxidized Vitamin C, Dehydroascorbic Acid, Reveals Its Functions *in vivo*

**DOI:** 10.1016/j.ebiom.2017.08.017

**Published:** 2017-08-22

**Authors:** Hongbin Tu, Yu Wang, Hongyan Li, Lauren R. Brinster, Mark Levine

**Affiliations:** a Molecular and Clinical Nutrition Section, Digestive Diseases Branch, Intramural Research Program, National Institute of Diabetes and Digestive and Kidney Diseases, National Institutes of Health, USA; b Division of Veterinary Research, National Institutes of Health, USA

**Keywords:** bromoAA, 6-deoxy-6-bromo-l-ascorbate, bromoDHA, 6-deoxy-6-bromo-dehydroascorbic acid, DHA, dehydroascorbic acid, GLUTs, glucose transporters, gulo^−/−^ mice, gulonolactone oxidase knockout mice, PBS, phosphate-buffered saline, RBCs, red blood cells, SVCT1, SVCT2, sodium-dependent vitamin C transporter, TCEP, tris(2-carboxyethyl)phosphine, WT, wildtype mice

## Abstract

Despite its transport by glucose transporters (GLUTs) *in vitro*, it is unknown whether dehydroascorbic acid (oxidized vitamin C, DHA) has any *in vivo* function. To investigate, we created a chemical transport knockout model using the vitamin C analog 6-bromo-ascorbate. This analog is transported on sodium-dependent vitamin C transporters but its oxidized form, 6-bromo-dehydroascorbic acid, is not transported by GLUTs. Mice (gulo^−/−^) unable to synthesize ascorbate (vitamin C) were raised on 6-bromo-ascorbate. Despite normal survival, centrifugation of blood produced hemolysis secondary to near absence of red blood cell (RBC) ascorbate/6-bromo-ascorbate. Key findings with clinical implications were that RBCs *in vitro* transported dehydroascorbic acid but not bromo-dehydroascorbic acid; RBC ascorbate *in vivo* was obtained only *via* DHA transport; ascorbate *via* DHA transport *in vivo* was necessary for RBC structural integrity; and internal RBC ascorbate was essential to maintain ascorbate plasma concentrations *in vitro*/*in vivo*.

## Highlights

• Red cells *in vivo* obtain vitamin C (ascorbate) by dehydroascorbic acid transport.
• Red blood cell ascorbate is necessary to maintain red blood cell structural integrity.
• Red blood cell ascorbate maintains external plasma ascorbate concentrations *in vivo* by transmembrane electron transfer.

In animals and humans, it is unknown whether the oxidized form of vitamin C, termed dehydroascorbic acid, has a physiologic purpose. Using a mouse model and a custom-synthesized vitamin C analog, we show that red blood cells obtain their vitamin C by transport of dehydroascorbic acid, instead of vitamin C itself. The transported material is reduced inside and has at least two physiologic functions. One is to maintain structural integrity of red blood cells, and the other is to maintain vitamin C in the liquid part of blood, plasma.

## 1 Introduction

Vitamin C (ascorbic acid, ascorbate) entry into cells is essential for all of its functions as a vitamin (Levine et al., 2011, Padayatty and Levine, 2016). Because ascorbate is charged at physiologic pH, it does not diffuse across membranes and requires transporters for cell entry. In cells and in expression systems utilizing *Xenopus* oocytes, two distinct transport mechanisms have been characterized. One is that ascorbate is transported as such, on sodium-dependent vitamin C transporters SVCT1 and SVCT2 (Tsukaguchi et al., 1999, Daruwala et al., 1999). A second mechanism is that ascorbate oxidizes to dehydroascorbic acid (DHA), which is transported on glucose transporters (GLUTs) (Vera et al., 1993, Washko et al., 1993, Rumsey et al., 1997, Corpe et al., 2013). Once intracellular, DHA is rapidly reduced to ascorbate. This mechanism has been termed ascorbate recycling (Washko et al., 1993, May et al., 1995).

It has been unclear what role, if any, DHA transport has in normal physiology and pathophysiology *in vivo*. Possible functions specific to DHA transport are worth understanding due to the structural similarity between glucose and DHA (Tolbert and Ward, 1982). Because DHA transport on GLUTs is competitively inhibited *in vitro* by glucose analogs (Rumsey et al., 1997), knowledge of DHA transport *in vivo* could have clinical implications in diabetes.

In experiments that could determine whether DHA transport was relevant, knockout mice for the sodium-dependent tissue transporter SVCT2 were created (Sotiriou et al., 2002). If mice utilized DHA transport for tissue accumulation, then DHA transport could rescue the absence of SVCT2, tissues of SVCT2 knockout mice could still contain ascorbate, and mice could appear normal. Alternatively, if DHA transport were specific to one or a few cell types, or unimportant *in vivo*, then SVCT2 knockout mice would be expected to be severely ascorbate deficient. These were the observed findings. SVCT2 knockout mice lacked ascorbate in all tissues measured, and did not survive more than minutes after birth.

One reasonable interpretation of SVCT2 knockout mouse experiments is that DHA transport followed by intracellular reduction was not physiologically relevant, at least in mice. If DHA had general physiologic relevance, then DHA transport should have prevented systemic tissue deficiencies and death. However, another explanation is that the cell type or tissue that utilized DHA transport was inadvertently not measured. Despite findings from SVCT2 knockout mice, there are several reasons to pursue DHA transport. DHA has equal or higher affinity than glucose for glucose transporters, such that hyperglycemia could inhibit DHA uptake and thereby create a link to diabetes and its complications (Rumsey et al., 1997, Corpe et al., 2013, Rumsey et al., 2000). Multiple redundant pathways exist in cells that immediately reduce DHA to ascorbate, utilizing both enzymatic and chemical reduction mechanisms (Maellaro et al., 1994, Winkler et al., 1994, Xu et al., 1996, Park and Levine, 1996, May, 2002, Padayatty and Levine, 2016). Existence of redundant pathways implies that DHA acid transport could have functional consequence(s) in as yet unidentified cell(s). In diabetes, if hyperglycemia could create ascorbate deficiency by locally inhibiting DHA transport in a specific cell type, this might uncover a localized heretofore unknown selective cellular deficiency in diabetes.

One possibility to investigate DHA specific transport pathways is to recapitulate the SVCT2 model, by creating knockout mice for DHA transporters. However, because these transporters are glucose transporters, primarily GLUT1 and GLUT3 (Rumsey et al., 1997, Rumsey et al., 2000, Corpe et al., 2013), their elimination would create overwhelming confounding variables. We chose an alternate path, by utilizing a compound that we had previously synthesized that could act as a chemical knockout for DHA transport. The goal was to test *in vivo* an ascorbate analog that was specific only for ascorbate transporters, and not transported by GLUTs. Ascorbate analogs were initially synthesized as 6-halo ascorbates, with 6 deoxy-6-bromo-l-ascorbic acid (bromoAA) as the working compound (Rumsey et al., 1999, Corpe et al., 2005). BromoAA was functionally tested using transporters expressed in microinjected *Xenopus laevis* oocytes and in cell models. BromoAA was transported only by SVCTs, with equal or higher affinity compared to ascorbate. When bromoAA was oxidized, 6-deoxy-6-bromo-dehydroascorbic acid (bromoDHA) formed but was not transported at all *in vitro* by GLUTs, in contrast to controls with DHA. The next step to determine function of dehydroascorbic acid transport, if any, was development of an *in vivo* system. Here, we describe findings in mice (gulo^−/−^ mice) unable to synthesize ascorbate that were provided exclusively with bromoAA.

## 2 Materials and Methods

### 2.1 Materials

BromoAA was synthesized as described (Rumsey et al., 1999, Corpe et al., 2005). Ascorbic acid was purchased from Sigma/Aldrich. DHA and BromoDHA were synthesized *de novo* from parent compounds immediately prior to experiments (Corpe et al., 2005, Li et al., 2012, Corpe et al., 2013). All other chemicals were highest purity grade obtainable commercially.

### 2.2 Mice and Tissue Samples From Mice

Animal experiments were approved by the Animal Care and Use Committee NIDDK, NIH, and were conducted in accordance with NIH guidelines. Mice were fed *ad libitum* on regular chow diet (NIH-07) without detectable ascorbate (detection limit 10 nM). Mice types were C57BL/6 (wildtype, WT) (Charles River Laboratories, Wilmington, MA, USA); gulonolactone oxidase (gulo^+/−^) mice (Mutant Mouse Regional Resource Center, University of California at Davis, USA), bred as described (Maeda et al., 2000). Homozygous gulo^−/−^ mice were bred from heterozygous gulo^+/−^ mice, and confirmed by genotyping using RT-PCR. If not stated otherwise, 8–12-week-old mice were used for experiments. Tissue samples were collected during pathological analysis. Tissue samples (≤ 100 mg) were harvested from mice and homogenized on ice in 100 μL (adrenal, pituitary) or 1000 μL (all other tissues) in ice-cold 90% methanol containing 1 mM EDTA. Samples were then centrifuged at 25,000*g* at 4 °C for 15 min. Supernatants were collected and diluted in 1:10 (heart) or 1:100 (adrenal gland, pituitary gland, small intestine, brain, liver, lung, and kidney) in 90% methanol containing 1 mM EDTA for ascorbate or bromoAA analyses. Pellets were diluted in 1 mL CHAPS for protein assay (Pierce). Mouse chow was analyzed for ascorbate in the same manner as tissue samples (Corpe et al., 2010).

Plasma and RBCs were collected from whole blood using centrifugation at 200* g* for 5 min at 4 °C to avoid hemolysis. When administered, mice received ascorbate or bromoAA supplements *via* drinking water at a dose of 1 g/L, and water was changed daily.

### 2.3 Histopathologic Examination

Mice (60–64 weeks old) were anesthetized, euthanized, and organs were then excised. The examined organs were brain, pituitary gland, liver, spleen, kidney, pancreas, heart, stomach, lung, small intestine, large intestine, limbs, spinal cord, eyes, ears, nose, tongue and salivary glands.

### 2.4 Confocal Microscopy

Confocal microscopy analyses of mouse RBCs were conducted as previously described (Tu et al., 2015). RBCs were fixed using 0.1% glutaraldehyde, stained with Alexa Fluor 488 phalloidin (5 units/mL), and analyzed using confocal microscopy at excitation/emission wavelengths of 489/518 nm. Whole cell and biconcave area diameters were measured using ZEN 2007 software by drawing a horizontal line across the center of the target RBC and calculating the distances between two marginal points.

### 2.5 Erythrocyte Osmotic Fragility

RBC osmotic fragility is a surrogate for RBC deformability (Clark et al., 1983). RBC osmotic fragility is based on resistance of RBCs to lysis as a function of decreasing NaCl concentrations and the assay was performed as described (Parpart et al., 1947) with modifications for mouse samples. Whole blood (150 μL) from each mouse was collected using heparinized Micro-Hematocrit capillary tubes (Fisher Scientific). NaCl solutions or distilled water (150 μL/well) were added to 12 wells of a 96-well round bottom plate. Concentrations of NaCl solutions were as 0.90%, 0.70%, 0.65%, 0.60%, 0.55%, 0.50%, 0.45%, 0.40%, 0.35%, 0.30% and 0.20%, one concentration per well. One 10 μL aliquot of whole blood was then added to the 12 wells containing NaCl solutions or distilled water. To avoid mechanical hemolysis, each well was gently mixed three times by pipetting up and down. Test plates were incubated for 60 min at room temperature, and subsequently centrifuged at 1740 * g* for 5 min at 4 °C. The resulting supernatant was transferred to a new 96-well flat bottom plate, and then hemoglobin content was determined at 540 nm using μQuant™ Microplate Spectrophotometer (Bio-Tek Instruments, Inc). Values from the well containing RBCs in 0.90% NaCl solution were used as blank. Values from the well containing RBCs in distilled water were used as 100%. Hemolysis status was presented as percentage: % Hemolysis = (O.D. of test well − O.D. of 0.90% NaCl well) / (O.D. of dH_2_O well − O.D. of 0.90% NaCl well) × 100%.

### 2.6 P50 Assay

The P50 value (pO_2_ at which 50% of hemoglobin is saturated with O_2_), parameter of the hemoglobin − oxygen dissociation rate, was determined by using a HEMOX Analyzer (TCS Scientific Co., New Hope, PA). Oxygen dissociation curves were graphed using dual wavelength spectrophotometry as described (Guarnone et al., 1995). Briefly, mouse whole blood (24 μL) was diluted in 4 mL of Hemox-solution (HS-500, pH 7.4 ± 0.01, TCS Scientific Co.) mixed with 8 μL anti-foam agent (AFA-25, TCS Scientific Co.). The resulting mixture was mixed and heated to 37 °C, and then oxygenated to 100% under air purging. Samples were subsequently deoxygenated under nitrogen purging. The P50 values were determined at the points of 50% oxygen saturation.

### 2.7 HPLC Analysis

Ascorbate was analyzed by reverse phase HPLC using a 5 μm, 250 × 4.6 mm ODS-DABS C18 (Ultrasphere 240,002; Beckman Coulter, Brea, CA, USA) with coulometric electrochemical detection as described previously (Li et al., 2012). BromoAA was also measured by HPLC, using the same detection system and settings but with a modification of the mobile phase methanol:water ratio to 47.5:52.5% (Corpe et al., 2005). Dehydroascorbic acid (DHA) was analyzed by reduction to ascorbate with tris(2-carboxyethyl)phosphine (TCEP) as described (Li et al., 2012).

Mice plasma and RBC samples were prepared with heparinized capillary tubes for HPLC analyses as described with minor modifications. Briefly, 60–80 μL of whole blood was obtained by mandibular puncture and flowed into hematocrit tubes by capillary action. Tubes were centrifuged at 200* g* for 5 min rather than 12,000 * g* for 2 min, to avoid mechanical hemolysis by centrifugation force.

### 2.8 Uptake of Ascorbate, DHA, and BromoDHA Into Mouse RBCs

To prepare DHA or bromoDHA, ascorbate or bromoAA (2 mM) was mixed with 2 μL bromine and purged with nitrogen until the brown color (bromine) disappeared, as described (Li et al., 2012). The product was further diluted to 100 μM for experimentation. RBCs from gulo^−/−^ mouse without ascorbate supplements for 4 weeks were washed three times using cold PBS or stop solution (PBS containing 20 μM cytochalasin B), and then incubated with 500 μL ascorbate, DHA, bromoAA, or bromoDHA (100 μM) at 0 °C or 37 °C. Stop solution (1 mL) was added at 0, 1, 2, 5 and 10 min to the RBC samples, and then samples were immediately kept on ice. The resulting RBCs were washed for three times using 1 mL stop solution at 4 °C. RBC ascorbate or bromoAA were extracted and measured using HPLC assay (Corpe et al., 2005, Li et al., 2012). For bromoDHA stability, bromoDHA (100 μM) was kept at 37 °C for 0–10 min before reducing to bromoAA by adding TCEP (0.5 mM final). BromoAA was measured using HPLC.

### 2.9 BromoAA Binding to Mouse RBCs Independent of Cytochalasin B

RBCs (60 μl) from unsupplemented gulo^−/−^ mice were incubated with 100 μM fresh bromoAA in the presence or absence of 20 μM cytochalasin B in PBS (0.5 mL) for 0, 1, 2, 5, 10, 30, 60, 90 and 120 min at 0 or 37 °C. Stop solution (PBS containing 20 μM cytochalasin B, 1 mL) was added at each time point. RBC bromoAA was measured as previously described using an HPLC assay (Corpe et al., 2005, Li et al., 2012).

### 2.10 BromoAA Binding to RBC Membranes

Unsealed ghosts from 200 μL of C57BL/6 WT mouse blood were prepared as described (Steck and Kant, 1974), and then 30 μL aliquots were incubated with 300 μL ascorbate or bromoAA in PBS (100 μM final concentrations) at 37 °C for 20 min. The ascorbate- or bromoAA-incubated ghosts were pelleted by centrifugation at 22,000*g* for 10 min. Ghost pellets were washed three times using 10 mL cold PBS at 4 °C and centrifuged at 18,000*g* for 10 min for each wash. Ascorbate and bromoAA were extracted with 90% methanol/1 mM EDTA and measured using HPLC (Li et al., 2012). Pellets were diluted in 1 mL CHAPS for protein assay (Pierce). Estimated concentrations of bromoAA were calculated as follows: ghosts were prepared from 200 μL mouse blood. Mouse hematocrit approximately 50%, therefore 100 μL packed RBCs. Internal volume is 70% (Mendiratta et al., 1998), and assuming 100% recovery, this is 70 μL. Total protein of prepared ghosts corresponding to this 70 μL was 26.5 μg. Measured bromoAA was 46.5 pmoles/μg protein. Therefore: 46.5 pmoles/μg × 26.5 μg protein = 1232 pmoles/70 μL = 17.6 μM. Estimated concentrations of ascorbate were calculated similarly.

### 2.11 Degradation of Plasma Ascorbate and BromoAA With or Without Mouse RBCs

Plasma and RBCs were separated from whole blood of wild type mice. RBCs were washed twice using ice cold PBS and adjusted to 50% hematocrit in PBS. Whole blood, plasma only, and RBCs only were incubated for 0, 0.5, 1, 2, 4 and 8 h at 15 °C. Plasma and RBC ascorbate concentrations were measured by HPLC at each time point as described previously (Li et al., 2012). Washed RBCs (50% hematocrit) were incubated with or without 500 μM TCEP for 0, 0.5, 1, 2 and 4 h, and intracellular and extracellular ascorbate was measured by HPLC analysis at each time point as described previously (Li et al., 2012). For blood samples of ascorbate deficient gulo^−/−^ mice, similar experiments were conducted at 15 °C, except that 30 μM ascorbate or bromoAA was added externally and samples were incubated for 2 h before analysis.

### 2.12 Ascorbate and BromoAA Efflux From Hepatocytes

Primary hepatocytes from C57BL/6 WT mice were isolated as described (Li et al., 2010). Cells were plated on 12 well plates at ~ 1 × 10^6^ cells / 2 mL/well and cultured over 24 h at 37 °C in a humidified atmosphere of 95% air and 5% CO_2_. Prior to efflux experiments, ascorbate or bromoAA 200 μM were preloaded three times to hepatic cells at time points of − 16 h, − 4 h, and − 1 h. Before each new preloading cells were washed twice with fresh medium. Immediately prior to efflux experiments, cells were washed three times with fresh media. To begin efflux, 400 μL fresh medium with 500 μM TCEP was added and cells were incubated for 30 min at 37 °C in a humidified atmosphere of 95% air and 5% CO_2_. Medium was then collected and centrifuged at 18,000*g* for 10 min. 400 μL of clear supernatant was mixed with 200 μL 90% methanol and 1 mM EDTA and processed for ascorbate and bromoAA analyses by HPLC with coulometric detection (Corpe et al., 2005, Li et al., 2012). Cell pellets were washed twice, intracellular ascorbate or bromoAA were extracted with 300 μL 90% methanol and 1 mM EDTA and measured in the same manner. Pellets obtained from cells after 90% methanol/1 mM EDTA precipitation were diluted in 1 mL CHAPS for protein assay (Pierce). Percentage of ascorbate or bromoAA released from primary liver cells (%) = ascorbate or bromoAA released / (ascorbate or bromoAA released + intracellular ascorbate or bromoAA).

### 2.13 Statistical Analysis

Data was presented as mean ± S.D. unless otherwise indicated. Number of mice and cells were included in the figure legends. Each data point represented at least three replicates. Points lacking error bars meant the error bars were smaller than the symbol sizes. Unless otherwise indicated, comparisons between three or more groups utilized one-way ANOVA followed by Tukey's multiple comparison test (SigmaPlot version 13). Unless otherwise indicated, significances were represented as follows: *p < 0.05, **p < 0.01, ***p < 0.001.

## 3 Results

**Fig. 1:**
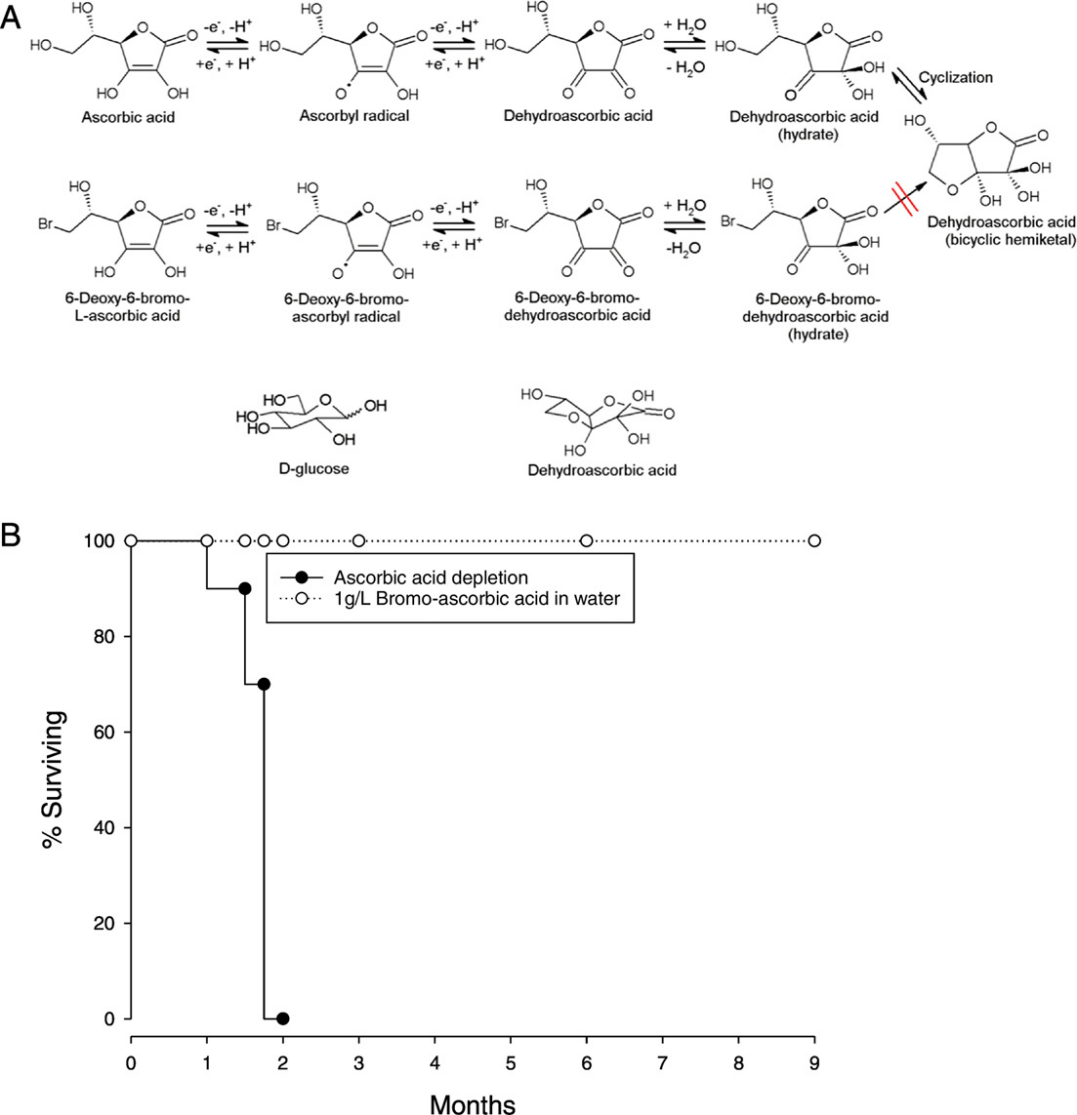
Bromoascorbate (BromoAA) as a chemical knockout for dehydroascorbic acid, and survival of gulo^−/−^ mice raised on bromoAA. A. Structural pathway of ascorbate oxidation to dehydroascorbic acid (DHA), and bromoascorbate (bromoascorbic acid, bromoAA) to dehydrobromoascorbic acid (bromoDHA). B. Kaplan-Meier survival curves of gulo^−/−^ mice (from 10 weeks old) depleted of ascorbate or supplemented with 1 g/L bromoAA in drinking water daily for 9 months. N = 10 mice per group.

With oxidation, ascorbic acid loses two electrons to form DHA. Upon water addition, DHA cyclizes to form a bicyclic hemiketal (Tolbert and Ward, 1982) (Corpe et al., 2005) (Fig. 1A, top line). When bromine replaces the hydroxyl group on the sixth carbon, the resulting compound is 6-deoxy-6-bromo-l-ascorbic acid (bromoAA). Oxidation of bromoAA yields 6-deoxy-6-bromo-dehydroascorbic acid (bromoDHA) as the product. However, the bromine substitution prevents cyclization (Fig. 1A, bottom line), in contrast to the consequences of water addition to DHA (Fig. 1A, top line). Both ascorbic acid and bromoAA are transported by sodium-dependent ascorbate transporters (SVCTs), which do not transport DHA (Tsukaguchi et al., 1999, Daruwala et al., 1999, Corpe et al., 2005). When ascorbate is oxidized to DHA, it is transported by facilitated glucose transporters, as DHA and glucose have similar structures (Fig. 1 bottom line) (Corpe et al., 2005). However, because bromoDHA does not cyclize, this compound is not transported on GLUTs (Corpe et al., 2005). Therefore, bromoAA is specific for ascorbate transport only. With bromoAA *in vivo*, bromoDHA transport by GLUTs should not occur, and functions of ascorbate that utilized only DHA transport could be revealed. Thus, if it were possible to replace AA with bromoAA *in vivo*, it could then be tested whether any ascorbate functions *in vivo* required DHA transport.

To determine whether DHA transport had *in vivo* relevance, we used gulonolactone oxidase knockout (gulo^−/−^) mice. These mice do not make ascorbate because the coding sequence of the terminal enzyme in the ascorbate biosynthetic pathway is intentionally mutated and not transcribed (Maeda et al., 2000). Immediately after weaning, these mice were raised for 9 months only on bromoAA. Gulo^−/−^ mice supplemented with bromoAA had normal survival (Fig. 1B), and minimal pathologic differences compared to gulo^−/−^ mice raised on ascorbate (Supplemental Table 1). Control gulo^−/−^ mice, raised without ascorbate and bromo AA after weaning, died after 6 weeks.

**Fig. 2:**
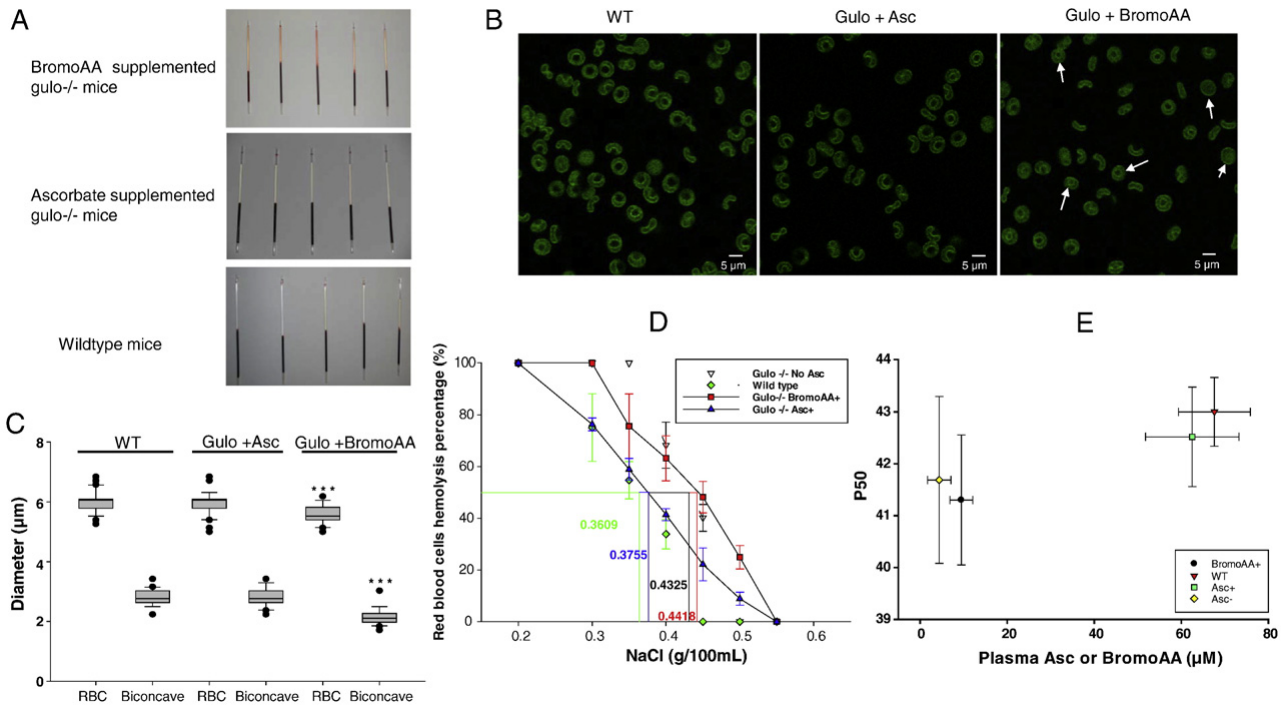
Characteristics of red blood cells (RBCs) obtained from gulo^−/−^ mice supplemented with bromoAA for 15 weeks. A. Centrifugation of mouse whole blood from unsupplemented wild type mice; gulo^−/−^ mice supplemented daily with 1 g/L bromoAA for 15 weeks; or gulo^−/−^ mice supplemented daily with 1 g/L ascorbate for 15 weeks. B. Confocal microscopy of RBCs from unsupplemented wild type mice and gulo^−/−^ mice supplemented with ascorbate or bromoAA. The ascorbate and bromoAA concentrations in plasma and RBCs were the same as shown in Fig. 3A. Examples of swollen RBCs are indicated by arrows. C. RBC and biconcave diameters of samples from wild type mice, gulo^−/−^ mice supplemented with ascorbate, or gulo^−/−^ mice supplemented with bromoAA. Thirty mouse RBCs in full view orientation were randomly selected from each group for measurement of cell diameters and biconcave area diameters. The diameters were determined using ZEN 2007 software (Carl Zeiss, Inc.) by drawing a horizontal line through the center of each target RBC and calculating the distances between two points where fluorescent intensities were most different (Tu et al., 2015). N = 30 RBC per point. *** P < 0.001. The ascorbate and bromoAA concentrations in plasma and RBCs were the same as shown in Fig. 3A. D. Osmotic fragility in mouse RBCs as a function of NaCl concentration. RBC samples were obtained from unsupplemented wild type mice; gulo^−/−^ mice supplemented with ascorbate or bromoAA for 15 weeks, and ascorbate depleted gulo^−/−^ mice. Ascorbate depleted mice were supplemented by gavage with 0.2 mg of ascorbate in 100 μL water once at 6 weeks. Gavage was performed to maintain mice at low ascorbate values while preventing demise from scurvy. N = 5 mice (starting from 18 weeks old) per point. The 50% lysis point for each condition (horizontal line) is determined by the vertical line of the same color. Statistics (two-tailed *t*-test) for 50% lysis: p < 0.001 for wildtype *vs* unsupplemented gulo^−/−^ mice, wildtype *vs* bromoAA gulo^−^/^−^ mice; p < 0.05 for unsupplemented *vs* ascorbate supplemented gulo^−/−^ mice, bromoAA *vs* ascorbate supplemented gulo^−^/^−^ mice. There were no statistical differences (p > 0.105) between wildtype and ascorbate supplemented gulo^−^/^−^ mice and unsupplemented *vs* bromoAA supplemented gulo^−^/^−^ mice. E. Oxygen dissociation (P50) values of RBCs from unsupplemented wild type mice (N = 5), gulo^−/−^ mice supplemented with ascorbate (N = 5) or bromoAA (N = 6), and ascorbate depleted gulo^−/−^ mice (N = 5). P = 0.12 for all groups (one way ANOVA); p = 0.04 for WT *vs* bromoAA (*t*-test).

Although gulo^−/−^ mice supplemented with bromoAA had no overt physical differences from ascorbate supplemented gulo^−/−^ mice, a phenotype emerged when whole blood was obtained to measure plasma ascorbate and bromoAA. Red blood cells (RBCs) hemolyzed when whole blood was centrifuged, using standard g force of 13,700, to separate plasma and RBCs (Fig. 2A). RBC appearance was examined using confocal microscopy. Cells appeared swollen, quantified by a decreased internal biconcave diameter (Fig. 2B,C). Hemolysis with this appearance is similar to what would be expected in hereditary spherocytosis clinically (Da et al., 2013). Patients with this disorder have RBCs that display osmotic fragility, as they are osmotically sensitive to decreasing sodium concentrations. Osmotic fragility is demonstrated when these RBCs lyse at higher sodium concentrations when compared to RBCs from healthy people. To display osmotic fragility, percentages of RBC lysis are plotted as functions of NaCl concentrations, and the NaCl concentration at which 50% of the RBCs lyse is quantitative measure of osmotic fragility. RBCs from mice raised on bromoAA had a right shifted osmotic fragility curve and a significantly shifted 50% lysis NaCl concentration, when compared to RBCs from gulo^−/−^ mice raised on ascorbate and to wildtype mice (Fig. 2D). Hereditary spherocytosis has similar osmotic fragility changes with fresh blood as performed here, and these changes are physiologically relevant (Eber et al., 1990). Oxygen-hemoglobin dissociation (p50) was measured in RBCs from WT mice; and from gulo^−/−^ without ascorbate, with ascorbate, and with bromoAA. Although P50 of RBCs from gulo^−/−^ mice raised on bromoAA differed from that of RBCs from wildtype mice (p < 0.04), when all groups were included, there were not significant differences (p = 0.12) (Fig. 2E).

**Fig. 3:**
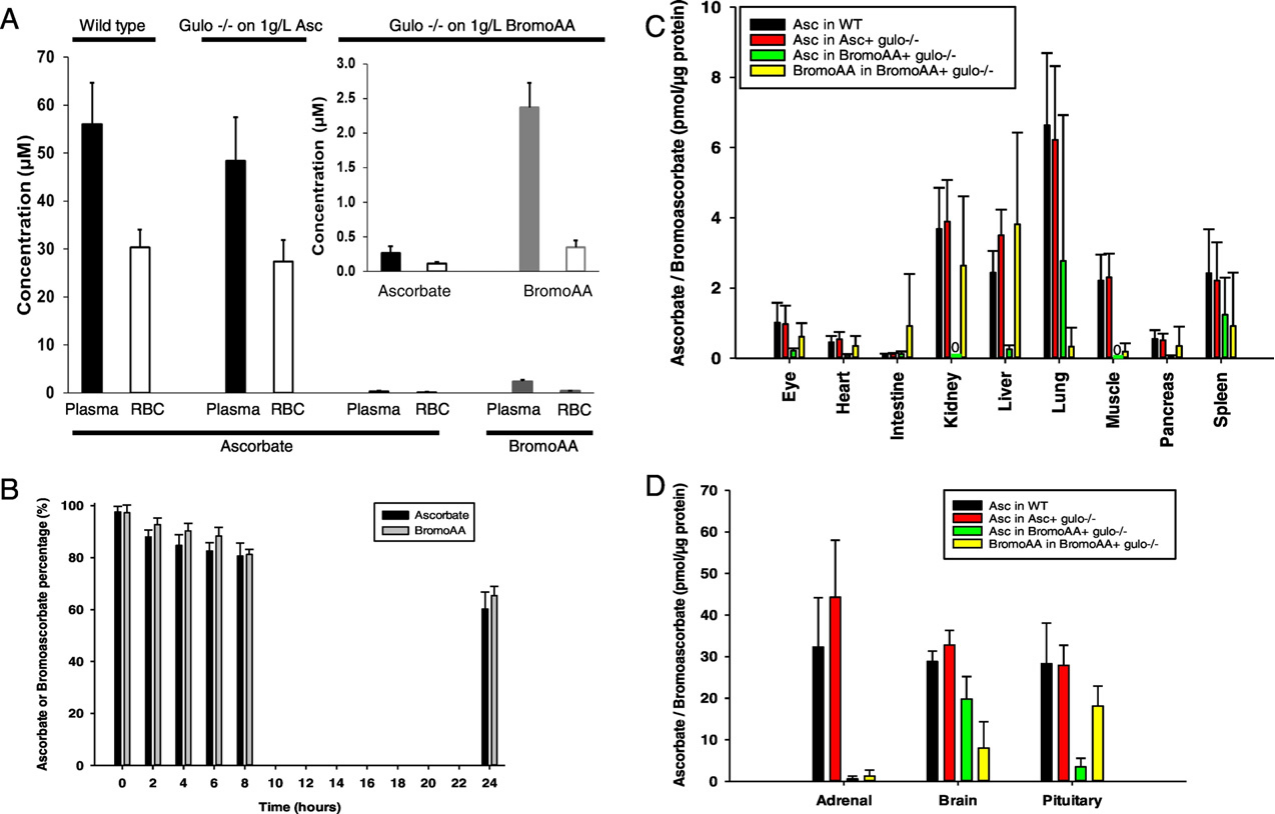
Ascorbate and bromoAA concentrations in gulo −/− mice. A. Ascorbate and bromoAA concentrations in plasma and RBCs from unsupplemented wildtype mice, gulo^−/−^ mice supplemented with ascorbate for 15 weeks, or gulo^−/−^ mice supplemented with bromoAA for 15 weeks. Inset: Expanded view of right side of Fig. 1A: plasma and RBC concentrations of ascorbate and bromoAA concentrations from gulo^−/−^ mice supplemented with bromoAA for 15 weeks. N = 5 mice per each point. Open bars indicate RBCs, closed bars indicate plasma. B. Ascorbate and bromoAA oxidation in drinking water. Ascorbate or bromoAA 1 g/L was added to drinking water and changed every 24 h. Water was sampled at 0, 2, 4, 6, 8, and 24 h to measure ascorbate or bromoAA. C, D. Tissue distribution of ascorbate and bromoAA in gulo^−/−^ mice supplemented with bromoAA for one year. Bars are: ascorbate in wildtype mice; ascorbate in ascorbate-supplemented gulo^−/−^ mice; ascorbate in bromoAA-supplemented gulo^−/−^ mice; bromoAA in bromoAA-supplemented gulo^−/−^ mice. Ascorbate or bromoAA levels are from tissues indicated that were isolated from gulo^−/−^ mice supplemented with bromoAA for one year (N = 7, 60–64 weeks old). Unsupplemented wild type mice (N = 5) and gulo^−/−^ mice supplemented with ascorbate (N = 5, 60–64 weeks old) were used as controls. Mouse tissues with low levels of ascorbate or bromoAA were grouped in C, including eye, heart, intestine, kidney, liver, lung, muscle, pancreas and spleen. Mouse tissues with high levels of ascorbate or bromoAA were shown in D, including adrenal gland, brain and pituitary gland. 0: not detected.

To characterize the hemolysis phenotype further, we measured bromoAA and ascorbate in plasma and RBCs. Plasma and RBC ascorbate were measured in two control groups: wild type mice, and gulo^−/−^ mice raised on ascorbate. Plasma ascorbate concentrations for controls were within an expected range, and were 56 μM and 49 μM respectively (Fig. 3A) (Tu et al., 2015). Similarly, RBC ascorbate concentrations for controls were within an expected range, and were 30 μM and 27 μM respectively (Fig. 3A) (Tu et al., 2015). In parallel, plasma and RBC bromoAA were measured in gulo^−/−^ mice raised on bromoAA. RBC bromoAA was as low as 0.4 μM, and plasma bromoAA was as low as 2.4 μM (Fig. 3A, inset). These low bromoAA concentrations could not be explained by preferential oxidation of bromoAA in drinking water, as oxidation rates were similar for bromoAA and ascorbate (Fig. 3B). The plasma and RBC findings for bromoAA each required further investigation.

**Fig. 4:**
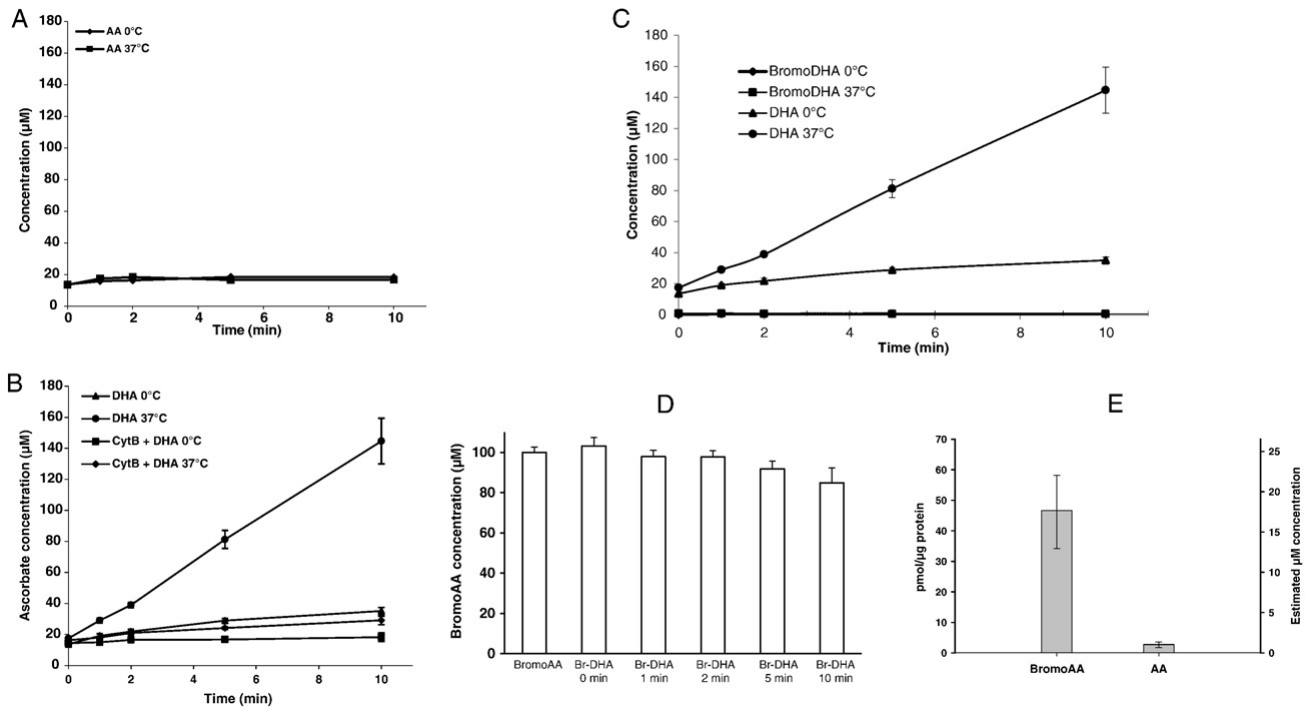
Ascorbate, dehydroascorbic acid (DHA) and bromoDHA transport into mouse RBCs. A. RBC ascorbate was measured in RBCs that were incubated with ascorbate (100 μM) for 0–10 min at 0 °C or 37 °C. B. Freshly prepared DHA (100 μM) was added for 0–10 min to RBCs at 37 °C with and without cytochalasin B pre-treatment and to RBCs at 0 °C with and without cytochalasin B pre-treatment. C. Freshly prepared bromoDHA (100 μM) was added for 0–10 min to RBCs at 37 °C and 0 °C; controls were freshly prepared DHA (100 μM) added for 0–10 min to RBCs at 37 °C and 0 °C. D. BromoDHA (100 μM) was kept for 0–10 min at 37 °C, and then reduced to bromoAA for measurement. E. BromoAA and ascorbate binding to unsealed ghosts of wildtype mouse RBCs. Unsealed ghosts prepared as in Methods from 200 μL of wildtype mouse RBC were incubated with 100 μM bromoAA or AA for 20 min at 37 °C. Ghost pellets were obtained by centrifugation, and ascorbate and bromoAA were measured by HPLC (n = 3).

To understand the low RBC bromoAA concentrations in gulo^−/−^ mice raised on bromoAA, transport mechanisms were explored. Control experiments were first conducted to validate how ascorbate entered RBCs (Tu et al., 2015). As predicted, no ascorbate transport was detected at 0 and 37 °C in the presence of 100 μM extracellular ascorbate (Fig. 4A). DHA was the transported substrate, and its transport was temperature-dependent (Fig. 4B). With 100 μM extracellular DHA for 10 min, intracellular ascorbate increased from 17 to 145 μM at 37 °C. Compared to this increase, DHA transport at 0 °C was 86% inhibited. Cytochalasin B, a GLUT inhibitor, prevented 80–90% of DHA transport at 37 °C and virtually 100% of transport at 0 °C (Fig. 4B). DHA uptake occurs when incubation media include physiological glucose concentrations (Tu et al., 2015). To test whether bromoDHA entered mouse RBCs, RBCs were incubated with 100 μM bromoDHA at 0 and 37 °C. No bromoAA was found in RBCs at either temperature (Fig. 4C). These findings could not be accounted for by inadvertent hydrolysis of extracellular bromoDHA during incubation, because extracellular bromoDHA was maintained during the experiments and was recoverable as bromoAA with reduction (Fig. 3B). To account for the low but not zero concentration of bromoAA associated with RBCs isolated from gulo^−/−^ mice raised on bromoAA (Fig. 3A inset), we hypothesized that bromoAA bound to RBC membranes due to increased molecular lipophilicity of bromine. To test this, we incubated mouse RBCs with 100 μM bromoAA in the presence or absence of 20 μM cytochalasin B at 37 °C. If bromoAA was able to utilize a GLUT transporter, cytochalasin B would inhibit entry. However, RBC bromoAA increased progressively over time independent of cytochalasin B (Supplemental Fig. 1A). To determine the maximal concentration of bromoAA on RBCs, we extended the incubation time to 2 h, and found RBC bromoAA achieved plateau at 17 μM after 1.5 h (Supplemental Fig. 1B). The *in vitro* ratio of RBC bromoAA: extracellular bromoAA was ~ 1/6, which was consistent with the *in vivo* ratio (Fig. 3A). These data suggest that bromoAA associated with RBCs was secondary to non-specific binding.

Next, it was tested directly whether bromoAA was bound to RBC membranes. Unsealed ghost preparations of wildtype mouse RBCs were prepared, and membranes were incubated with either bromoAA or ascorbate (Fig. 4E). Although ascorbate binding was minimal, bromoAA binding was approximately 10-fold higher, consistent with non-specific binding.

To understand the low plasma bromoAA in gulo^−/−^ mice raised on bromoAA, additional mechanisms were explored. Because bromoAA was orally provided by supplemented drinking water, several factors involved in absorption were considered. In the drinking water, bromoAA oxidized at the same rate as ascorbate (Fig. 3B). Ascorbate transporters in mouse intestine showed similar activity of transporting bromoAA as ascorbate (Corpe et al., 2010). BromoAA in the majority of tissues from gulo^−/−^ mice raised on bromoAA were similar to ascorbate in gulo^−/−^ mice controls raised on ascorbate (Fig. 3C,D). Taken together, these observations suggest that bromoAA and ascorbate behaved similarly during absorption.

A reasonable hypothesis to account for the low plasma bromoAA concentrations is based on what has been proposed to occur in plasma with ascorbate: a form of ascorbate recycling (Washko et al., 1993, Mendiratta et al., 1998). Utilizing transmembrane single electron transfer, ascorbate within RBCs could transfer an electron to extracellular, or plasma, ascorbate radical, which would have been generated by the first step of oxidation of plasma ascorbate. With dismutation of 2 molecules of ascorbate radical, one molecule of dehydroascorbic acid would form, which would then be transported back into RBCs on GLUTs and undergo immediate reduction. If this explanation were correct, we predicted that with bromoAA rather than ascorbate in plasma, plus minimal ascorbate in RBCs, plasma bromoAA concentrations could not be maintained. Thus, we investigated whether RBCs under different conditions were necessary to maintain plasma ascorbate.

**Fig. 5:**
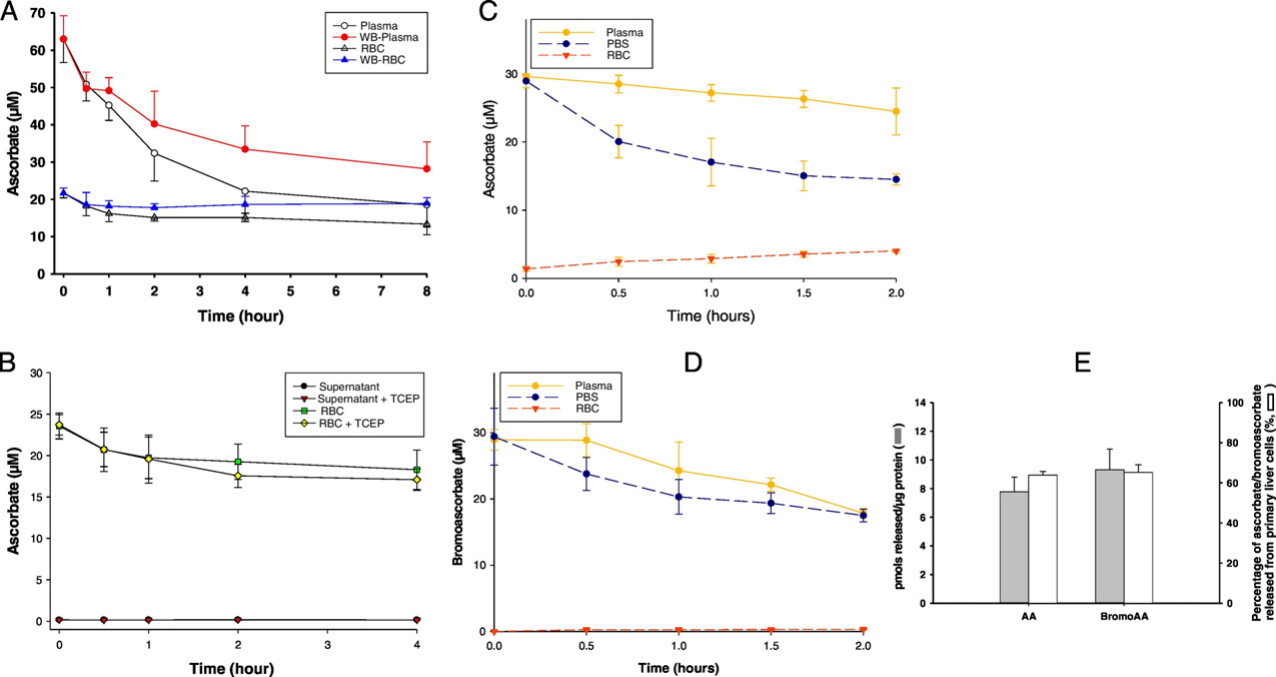
Maintenance of plasma ascorbate or plasma bromoAA by RBC ascorbate. A. Ascorbate degradation in plasma or RBCs from WT mice, n = 3 mice per group. Plasma: Plasma only; WB-plasma: plasma from whole blood; RBC: RBCs only; WB-RBC: RBCs from whole blood. B. Ascorbate efflux from RBCs from WT mice. n = 3 mice per group. Supernatant: supernatant from 50% hematocrit RBCs; supernatant + TCEP: supernatant from 500 μM TCEP final concentration and 50% hematocrit RBCs; RBC: RBCs from 50% hematocrit RBCs; RBC + TCEP: RBCs from 500 μM TCEP plus 50% hematocrit RBCs. C. Recovery of ascorbic acid (30 μM) spiked into plasma with or without 50% RBCs from ascorbate deficient gulo^−/−^ mice, n = 3 mice per group. Plasma: plasma from 30 μM ascorbate-spiked plasma plus 50% RBCs; PBS: plasma from 30 μM ascorbate-spiked plasma plus PBS (equivalent volume of 50% RBCs); RBC: RBCs from 30 μM ascorbate-spiked plasma plus 50% RBCs. D. Recovery of bromoAA (30 μM) spiked into plasma with or without 50% RBCs from ascorbate deficient gulo^−/−^ mice, n = 3 mice per group. Plasma: plasma from 30 μM bromoAA-spiked plasma plus 50% RBCs; PBS: plasma from 30 μM bromoAA-spiked plasma plus PBS (equivalent volume of 50% RBCs); RBC: RBCs from 30 μM bromoAA spiked plasma plus 50% RBCs. E. Release of bromoAA and ascorbate from hepatocytes. Isolated primary mouse hepatocytes were preloaded three times over 16 h with ascorbate or bromoAA. After washing and 30-min incubation in media without either compound added, intracellular and released ascorbate or bromoAA were measured. Percent release was calculated as described in Methods, n = 3.

In control experiments utilizing wildtype mice, samples of whole blood, plasma only, and washed RBCs only were incubated for up to 8 h at 15 °C, and ascorbate was measured in all samples at each time point (Fig. 5A). Ascorbate loss in plasma was approximately 50% greater without RBCs compared to plasma with RBCs. Although proposed by others (Montel-Hagen et al., 2008), ascorbate and dehydroascorbic acid did not efflux from RBCs, as no external ascorbate was detected even when a reducing agent was present (Fig. 5B). To further test the need of RBC ascorbate to stabilize plasma ascorbate, ascorbate depleted gulo^−/−^ mice were used that had not received ascorbate for 10 weeks, such that plasma and RBC ascorbate were ≤ 2 μM. To whole blood obtained from these mice, additions were made *in vitro* with either 30 μM ascorbate (Fig. 5C) or 30 μM bromoAA (Fig. 5D). Samples of whole blood, plasma only, and washed RBCs only were incubated for up to 2 h. For ascorbate experiments, plasma ascorbate loss was minimal when RBCs were present, and increased RBC ascorbate accounted for much of the loss, as predicted. Without RBCs, plasma ascorbate declined by nearly ½ of the initial value at 2 h (Fig. 5C). When whole blood from ascorbate depleted gulo^−/−^ mice was obtained and 30 μM bromoAA was added *in vitro*, the findings were quite different (Fig. 5D). BromoAA was lost from plasma whether or not RBCs were present, and RBC ascorbate was nearly undetectable and did not increase. Together these findings are consistent with the concept that ascorbate within RBCs maintains plasma ascorbate. Because bromoDHA cannot enter RBCs and is not internalized as bromoAA within RBCs, plasma bromoAA was not maintained *in vitro*, consistent with *in vivo* findings (Fig. 3A).

Ascorbate is released from hepatocytes, and presumably hepatocyte release is the source of nearly all ascorbate in animals that synthesize the vitamin (Upston et al., 1999). Thus, an alternate explanation of low plasma bromoAA concentrations found in mice raised on bromoAA is differential release of bromoAA and ascorbate from hepatocytes. This possibility was tested in primary hepatocytes. After isolation, primary hepatocytes were pre-loaded with ascorbate or bromoAA. After washing, percent released at 30 min for each compound was determined, and was indistinguishable (Fig. 5E). These findings dovetail with those indicating that RBC ascorbate maintains plasma ascorbate.

BromoAA toxicity is unlikely explanation of the findings, as pathologic differences were minor in mice raised on bromoAA for 9 months compared to control gulo^−/−^ mice raised on ascorbate. Plasma and RBC values from these mice (Supplemental Fig. 2) were similar to those found from shorter 4 month experiments (Fig. 3A). As an additional test of possible bromoAA toxicity, we investigated whether hemolysis was specific to bromoAA treated mice, compared to gulo^−/−^ mice that had not received ascorbate for 10 weeks. Plasma bromoAA and ascorbate values were similar and were < 5 μM, and RBC hemolysis was similar in both groups of mice (Fig. 2A, Supplemental Fig. 3). We conclude that absence of ascorbate, rather than bromoAA toxicity, explains hemolysis.

## 4 Discussion

In this paper, we used bromoAA to replace ascorbic acid in gulo^−/−^ mice that are unable to synthesize the vitamin. The oxidation product of bromoAA, bromoDHA, is not transported at all on glucose transporters, in contrast to the ascorbate oxidation product DHA. BromoAA serves as a chemical knockout for DHA transport, thereby allowing us to test whether DHA transport had specific functions *in vivo*. Although gulo^−/−^ mice had normal survival when they were raised on bromoAA, an *in vivo* function of DHA was revealed when plasma and RBC measurements were performed. First, RBCs obtained from these mice hemolyzed with centrifugation, and bromoAA within RBCs was nearly undetectable, suggesting that DHA was the only means whereby RBCs acquired vitamin C *in vivo*. Second, when gulo^−/−^ mice were raised on bromoAA, plasma concentrations of bromoAA were surprisingly low at < 3 μM, even though the material was stable in drinking water and is absorbed by mice (Corpe et al., 2010). Coupled to experiments with isolated RBCs using ascorbate and bromoAA, these data support the concept that electrons from internal RBC AA are normally utilized to maintain plasma ascorbate *via* transmembrane electron transfer (Orringer and Roer, 1979, Grebing et al., 1984, Su et al., 2006). DHA entry into RBCs is linked to the process, and the process was aberrant in gulo^−/−^ mice raised on bromoAA.

The phenotype of hemolysis in gulo^−/−^ mice raised on bromoAA was secondary to near absence of bromoAA and ascorbate in RBCs. These findings were supported by the complete inability of RBCs to transport bromoDHA *in vitro*. The findings could not be due to inability of bromoDHA to be reduced, as bromoAA was nearly fully recovered by reduction (Fig. 4D) (Corpe et al., 2005). RBCs from gulo^−/−^ mice that had not been fed bromoAA and from which ascorbate had been withheld had low ascorbate concentrations. These RBCs also hemolyzed with centrifugation, indicating that hemolysis was not due to toxicity from the bromine moiety. Detailed characterization of RBC hemolysis due to low internal ascorbic acid concentrations has recently been described elsewhere (Tu et al., 2015).

Two findings from experiments with gulo^−/−^ mice raised on bromoAA merit comment. Although bromoAA concentrations within RBCs were low as 500 nM, material was still detectable. Most likely, this was due to non-specific binding of bromoAA to RBCs, as demonstrated *in vitro* (Fig. 4E, Supplemental Fig. 1A, B). Unexpectedly, gulo^−/−^ mice raised on bromoAA had low but detectable (nanomolar) concentrations of ascorbate in both plasma and RBCs both at 15 weeks and at 9 months. Gulo^−/−^ mice could not make ascorbate, as unsupplemented gulo^−/−^ mice all died within 2 months. Gulo^−/−^ mice also did not receive any ascorbate in their food, as ascorbate was not detectable at all in mouse chow. Ascorbate was not detectable in mouse bedding, and synthesized bromoAA had no ascorbate contamination. The most likely explanation of low ascorbate concentrations in gulo^−/−^ mice raised on bromoAA is that these mice were able to metabolize the bromo moiety, with the result that low ascorbate concentrations were produced.

Gulo^−/−^ mice raised on bromoAA for 9 months had normal survival and minimal pathologic changes, compared to gulo^−/−^ mice raised on ascorbate. Survival may have been secondary to bromoAA, reduced but detectable ascorbate concentrations, or both. Survival on bromoAA is consistent with the findings that bromoAA can substitute for ascorbate in processes necessary for life. BromoAA reversed scurvy in guinea pigs (Kasai et al., 1995), had a lower Km for prolyl hydroxylase than ascorbate (Tschank et al., 1994), and was more efficiently transported by sodium-dependent ascorbate transporters than ascorbate (Corpe et al., 2005). BromoAA as well as 6-chloro-6-deoxy ascorbate transferred an electron to the oxidizing radicals with similar rate constants to ascorbate, indicating that one-electron reduction potential of these halo-ascorbates is similar to that of ascorbate (Bonifacic et al., 1994). Similarly, oxidation kinetics for 6-fluoro-6-deoxy ascorbate are similar to those of ascorbate (Madaj et al., 2000).

We investigated whether internal RBC ascorbate modulated oxygen dissociation from hemoglobin. Although p50 of RBCs from mice raised on bromoAA differed from wildtype mice, no significant differences were found when all groups of mice were compared. The p50 for mouse RBCs is higher than that for humans. Surprisingly, there is minimal information about ascorbate interactions with hemoglobin (Horejsi and Komarkova, 1960). Lack of prior data may have been due to difficulty in measuring RBC ascorbate, with only recent availability of a specific and sensitive assay (Li et al., 2012). Clinical relevance of potential ascorbate hemoglobin interactions in humans deserves to be explored. Recent evaluation of chelation therapy in patients with diabetes indicated that chelation therapy was as good as a first line diabetes medication in delaying vascular complications of diabetes (Lamas et al., 2013, Lamas and Ergui, 2016). Although the findings were attributed to chelation therapy itself, intravenous ascorbic acid is part of chelation therapy. The pharmacologic dose of ascorbic acid used, 7 g administered over 3 h, is enough to elevate plasma and presumably RBC ascorbate concentrations (Padayatty et al., 2004, Li et al., 2012). Right shifted p50 as a consequence of elevating ascorbate within RBCs dosing is one potential mechanistic explanation of the results, as is ascorbate-induced changes to RBC structural proteins such as β-spectrin (Tu et al., 2015). Experiments with human RBCs are indicated to explore whether a range of ascorbate concentrations, from oral and/or intravenous dosing, can change p50 and/or RBC structural proteins in RBCs from healthy humans and humans with diabetes.

Although not intuitive, it has been known for many years that plasma isolated from whole blood at 4 °C is more stable than plasma stored at 4 °C without RBCs (Dhariwal et al., 1991). There have been two hypotheses concerning how RBCs maintain plasma ascorbate. One is that there is direct efflux of ascorbate/dehydroascorbic acid from within RBCs (Montel-Hagen et al., 2008). The findings here and elsewhere (May et al., 2000) do not support this hypothesis. The second hypothesis is based on ascorbate recycling, described in neutrophils and in RBCs (Washko et al., 1993, Mendiratta et al., 1998). For human RBCs, the proposed mechanism is that as ascorbate within RBCs oxidizes to ascorbate radical, an electron is transferred to a transmembrane protein cytochrome b561 (Su et al., 2006). The electron carried on cytochrome b561 is directed outward and reduces external (plasma) ascorbate radical that forms as the first step of plasma ascorbate oxidation. Reduction of external ascorbate radical to ascorbate would stabilize ascorbate in plasma. Ascorbate radical reduction is not 100% efficient, as both humans and gulo^−/−^ mice develop scurvy with ascorbate withdrawal. Two molecules of ascorbate radical undergo dismutation, with formation of one ascorbate molecule and one DHA molecule. DHA is then transported into RBCs and reduced to ascorbate, providing partial replacement of ascorbate that oxidized. Because bromoDHA does not enter RBCs, bromoAA would be predicted to disrupt ascorbate recycling, such that plasma bromoAA would not be maintained. These were the observed findings, both *in vitro* and *in vivo*. Indeed, the data here with gulo^−/−^ mice raised on bromoAA provide firm evidence that ascorbate recycling occurs *in vivo*.

**Fig. 6:**
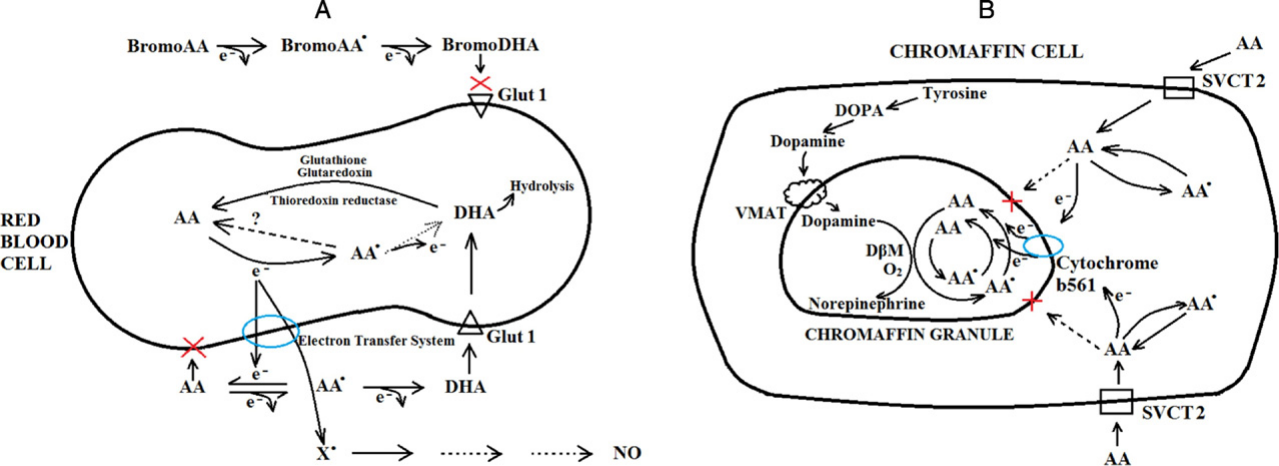
Ascorbate recycling by red blood cells (A) and chromaffin granules (B). A. A transmembrane electron transfer system transfers electrons from cytosolic ascorbate to extracellular ascorbate radical. Plasma (extracellular) ascorbate loses one electron to form ascorbate radical (AA) and a second electron to form dehydroascorbic acid (DHA). DHA is transported on GLUT 1 into the red cell and immediately reduced by glutathione, glutaredoxin, or thioredoxin reductase to ascorbate. Ascorbate within the red blood cell provides an electron to a putative transmembrane electron transfer system. The electron undergoes transmembrane transfer to an external receptor, either ascorbate radical or an unknown electron acceptor (X^.^) that might drive nitric oxide (NO) formation. Abbreviations: AA, ascorbate; AA^.^, ascorbate radical; BromoAA, bromo-ascorbate; BromoAA^.^, bromo-ascorbate radical; BromoDHA, bromo-dehydroascorbic acid; DHA, dehydroascorbic acid; GLUT1, glucose transporter 1; NO, nitric oxide; X^.^, (unknown) external electron acceptor for transmembrane electron transfer. B. Cytochrome b561 transfers electrons from cytosolic ascorbate to ascorbate radical in the chromaffin granule. Dopamine formed in cytosol from tyrosine is transported into the chromaffin granule by the vesicular mono-amine transporter (VMAT). The enzyme dopamine β-monooxygenase (DßM) + plus two electrons are needed for norepinephrine biosynthesis. Single electrons are provided by ascorbic acid (AA) within chromaffin granules with formation of ascorbate radical (AA^.^). AA^.^ is reduced back to AA by electrons transferred from cytosolic AA to cytochrome b561 on the chromaffin granule membrane. AA is transported into cytosol by the AA transporter SVCT2, but AA cannot enter the chromaffin granule. Abbreviations: AA, ascorbate; AA^.^, ascorbate radical; DßM, dopamine β-monooxygenase; SVCT2, sodium-dependent vitamin C transporter 2; VMAT, vesicular mono-amine transporter.

Mature mouse RBCs do not have detectable cytochromeb561 (Su et al., 2006). Although unlikely, one explanation is that synthetic peptides used to make cytochrome antibodies were not active against epitopes in mouse RBCs. A more compelling explanation is that an NADH-dependent ascorbate reductase activity that has been described in RBCS accounts for the findings observed here (Orringer and Roer, 1979, Grebing et al., 1984, Marques and Bicho, 1997, Zamudio et al., 1969). Fig. 6A displays a scheme of RBC transmembrane electron transfer for maintenance of plasma ascorbate, and inability of bromoDHA to participate as an electron donor.

Ascorbate transmembrane electron transfer was originally described in adrenal medullary secretory vesicles, chromaffin granules, and is a key component of norepinephrine biosynthesis (Wakefield et al., 1986, Dhariwal et al., 1989, Fleming and Kent, 1991, Njus et al., 2001) (Fig. 6B). In brief, dopamine, the precursor of norepinephrine, is transported into chromaffin granules by a vesicular monoamine transporter on the chromaffin granule surface. The intragranular enzyme dopamine β-monooxygenase mediates norepinephrine biosynthesis utilizing internalized dopamine plus intragranular ascorbate as a true co-substrate. To synthesize norepinephrine from dopamine, oxygen plus two electrons are required. Each electron comes from one intragranular ascorbate, with formation of ascorbate radical. Extragranular (cytosolic) ascorbate transfers an electron to cytochrome b561 on the external side of a chromaffin granule. Cytochrome b561 transfers an ascorbate-derived electron to the intragranular side of the chromaffin granule, and reduces ascorbate radical back to ascorbate. In this manner, extragranular ascorbate provides the electrons for intragranular norepinephrine biosynthesis by transmembrane electron transfer. Extragranular ascorbate is oxidized, and intragranular ascorbate is maintained. The chromaffin granule is impermeant to ascorbate itself, and only single electrons from ascorbate are transferred. It is unknown how ascorbate itself enters chromaffin granules: possibilities include trapping during granule assembly, and entry as DHA.

For the RBC, it appears that electron transfer is inward to outward, but the mechanistic principles may be similar. In chromaffin granules, the only acceptor of electrons from cytochrome b561 is ascorbate radical. For electrons from ascorbate within RBCs that are directed outward, it is unknown whether there are other electron acceptors in addition to ascorbate. Although controversial, it has been proposed that nitric oxide formation by RBCs in relation to hypoxia could mediate vasodilatation (Kleinbongard et al., 2006, Bunn et al., 2010). The data here raise the possibility that electrons from ascorbate within RBCs are a source of synthesis or maintenance of plasma nitric oxide concentrations, through one or more intermediate extra-RBC electron acceptors other than ascorbate. Such intermediates may be worth identifying as they may vary in relation to ascorbate status in disease.

Despite the limitation of the bromoAA gulo^−/−^ mouse model, in that mice maintained minimal plasma and lowered tissue ascorbate concentrations, the model revealed three key findings. First, an essential function of DHA is coupled to its transport into RBCs, followed by subsequent immediate reduction. Second, RBC ascorbate is necessary to maintain RBC structural integrity. Third, internal RBC ascorbate maintains external ascorbate plasma concentrations *in vivo*. The chemical knockout model with bromoAA serves as a foundation for linking ascorbate, RBCs, and diabetes (Tu et al., 2015). This is because of the potential of the excess glucose concentrations that occur in diabetes to compete with DHA transport. Whether transmembrane electron transport from internal to external ascorbate is disrupted in diabetes, and whether there are clinical consequences, are logical avenues worth pursuing, because ascorbate concentrations are easily increased by dietary supplementation in patients who have low values.

## Appendix A Supplementary data

Supplementary figure and table legends. Image 1

Supplementary figures and table. Image 2
