# Supplementary material for: Chemical Transport Knockout for Oxidized Vitamin C, Dehydroascorbic Acid, Reveals Its Functions in vivo
Source: eBioMedicine. 2017 Aug 22;23:125–35. doi: 10.1016/j.ebiom.2017.08.017 (PMC5605377; doi:10.1016/j.ebiom.2017.08.017)
Supplement: Supplementary file 1 — Supplementary figure and table legends. [file mmc1.docx]

**Supplemental Figure Legends**

**Supplemental Fig. 1.** BromoAA binds to mouse RBCs independent of cytochalasin B. A. RBCs (
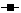
) and cytochalasin B-treated RBCs (
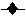
) were incubated with bromoAA (100 μM) for 0-10 min at 37°C, and then RBC bromoAA was measured. B. RBCs were incubated with bromoAA (100 μM) for 0-120 min at 0°C (
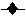
) or 37°C (
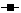
), and then RBC bromoAA was measured.

**Supplemental Fig. 2.** Ascorbate or bromoAA levels in plasma and RBCs from gulo^-/-^ mice supplemented with bromoAA for one year (N = 10, 60-64 weeks old). Plasma (
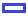
) or RBC (
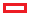
) ascorbate in unsupplemented wild type mice (N = 5, 60-64 weeks old) and gulo^-/-^ mice supplemented with ascorbate for one year (N = 5, 60-64 weeks old) were used as controls.

Supplemental Fig. 3. **Mechanical hemolysis and ascorbate plasma concentrations in gulo^-/-^ mice with or without ascorbate supplements.**

**Supplemental Table 1. Histo**pathologic findings of gulo^-/-^ mice supplemented with bromoAA (N = 7, 60-64 weeks old) or ascorbate (N = 3, 60-64 weeks old) for one year.
