# Supplementary material for: Chemical Transport Knockout for Oxidized Vitamin C, Dehydroascorbic Acid, Reveals Its Functions in vivo
Source: eBioMedicine. 2017 Aug 22;23:125–35. doi: 10.1016/j.ebiom.2017.08.017 (PMC5605377; doi:10.1016/j.ebiom.2017.08.017)
Supplement: Supplementary file 2 — Supplementary figures and table. [file mmc2.pdf]

Supplemental Figure 1

A

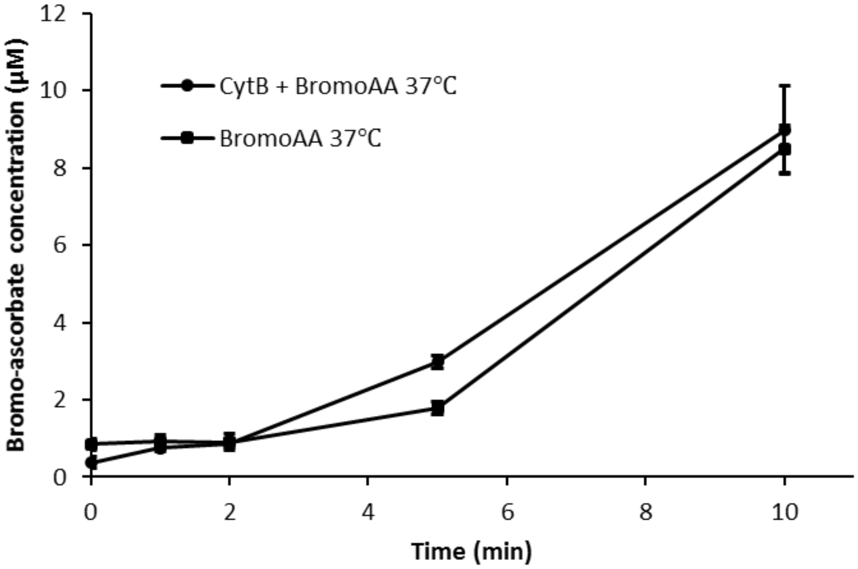

B

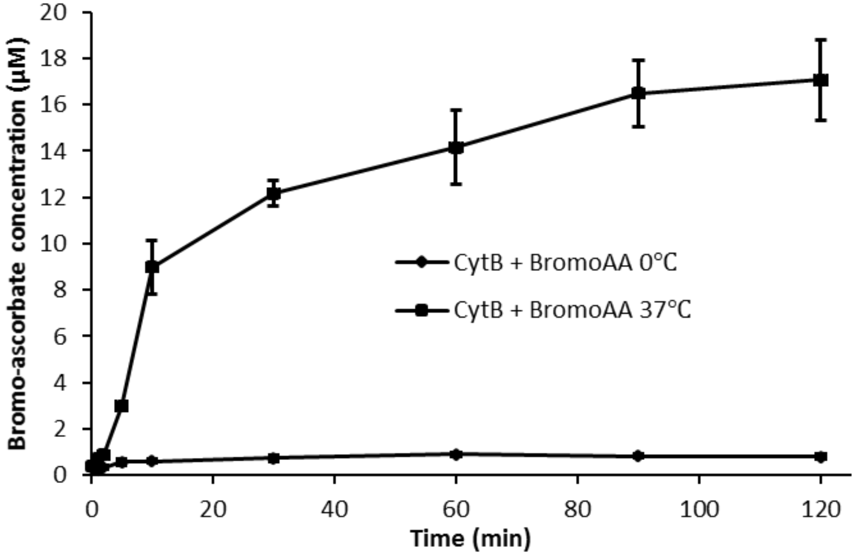

Supplemental Figure 2

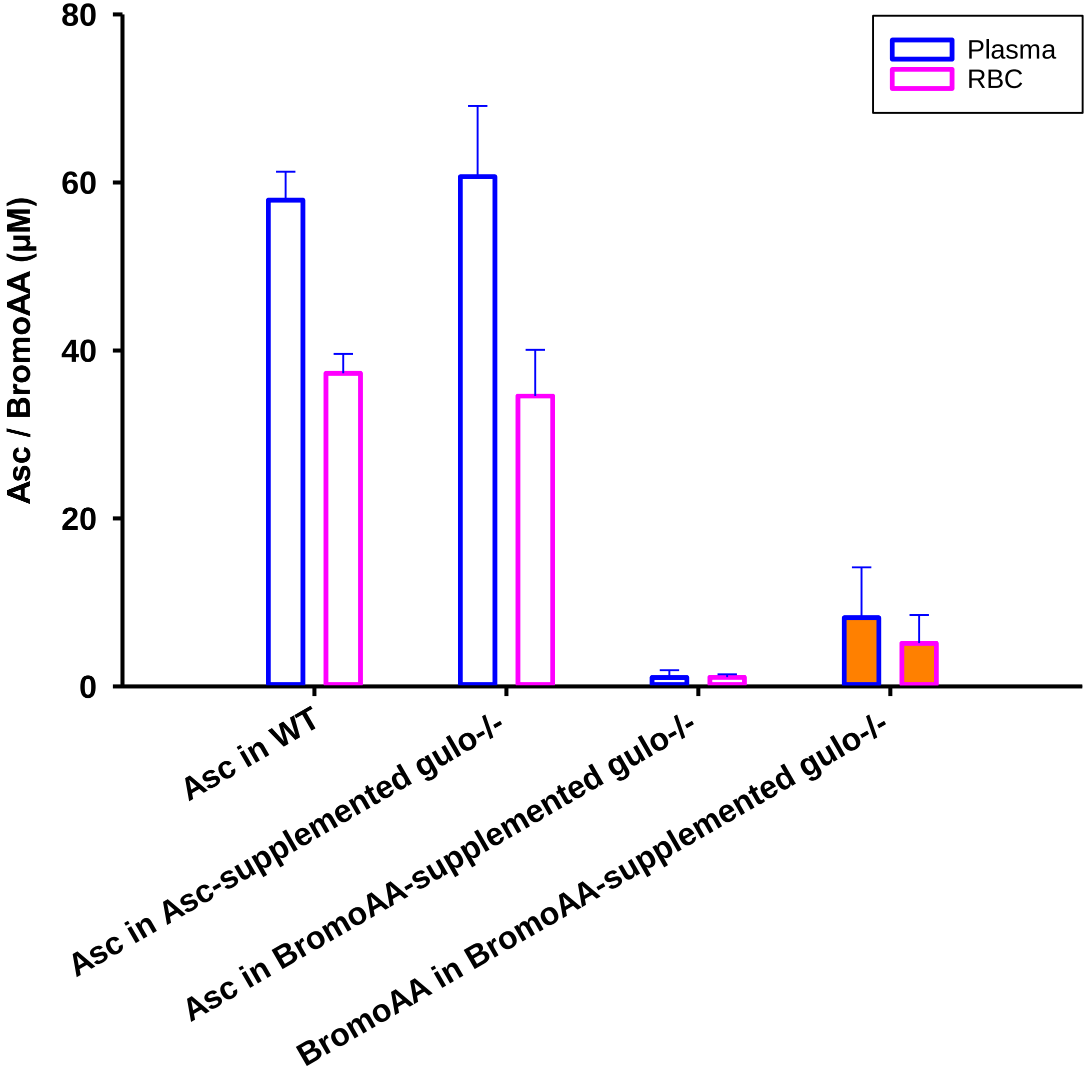

Supplemental Figure 3

Ascorbate Supplemented

Unsupplemented

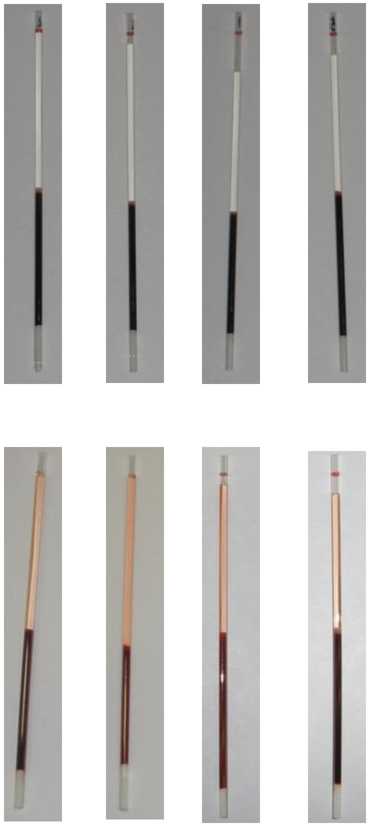

Ascorbate 62.5 ± 9.8 µM

Ascorbate 4.4 ± 2.7 µM

Supplemental Table 1 Histopathologic findings of gulo$^{-/-}$ mice supplemented with bromoAA (N = 7, 60-64 weeks old) or ascorbate (N = 3, 60-64 weeks old) for one year

BrAA gulo-/- mice		AA gulo-/- mice	
A	Ear (middle and inner) – Spiral ganglion atrophy, Spiral ligament degeneration; Kidney, Salivary glands – lymphoid aggregates	63	Salivary glands – exorbital dacryoadenitis; Liver – Hepatitis; Harderian gland – lymphoid aggregates
B	Ear (middle and inner) – Spiral ganglion atrophy, Spiral ligament degeneration; Kidney, Salivary glands, Liver, Lung – lymphoid aggregates; Harderian gland – adenomas; Liver – Hepatitis	64	Ear (middle and inner) – polyps, mineralization and osseous metaplasia; Salivary glands – exorbital dacryoadenitis, lymphoid hyperplasia; Kidney – lymphoid aggregates, tubular proteinosis; Liver – Hepatitis
C	Ear (middle and inner) – Spiral ganglion degeneration; Kidney, Salivary glands, Lung – lymphoid aggregates; Liver – Hepatitis; Harderian gland – adenomas	65	Salivary glands – exorbital dacryoadenitis, Kidney – lymphoid aggregates, tubular proteinosis; Liver – Hepatitis
D	Ear (middle and inner) – Spiral ganglion degeneration; Kidney, Salivary glands, Liver – lymphoid aggregates; Liver – Hepatitis; Harderian gland – adenomas		
E	Ear (middle and inner) – Spiral ganglion degeneration; Kidney, Salivary glands, Harderian gland – lymphoid aggregates; Liver – Hepatitis		
F	Ear (middle and inner) – Spiral ganglion, Spiral ligament degeneration; Kidney, Salivary glands, Liver, Pancreas – lymphoid aggregates; Harderian gland – adenomas		
G	Ear (middle and inner) – Spiral ganglion, Spiral ligament degeneration; Kidney, Salivary glands – lymphoid aggregates; Liver – Hepatitis; Harderian gland – adenomas		
